# Does an increase in serum FGF21 level predict 28-day mortality of critical patients with sepsis and ARDS?

**DOI:** 10.1186/s12931-021-01778-w

**Published:** 2021-06-22

**Authors:** Xing Li, Hua Shen, Tinghong Zhou, Xiaoyu Cao, Ying Chen, Yan Liang, Ting Lu, Jiafen He, Zhoulin Dou, Chuankai Liu, Yong Tang, Zexiang Zhu

**Affiliations:** Department of Critical Care, Changsha of Traditional Chinese Medicine Hospital, No. 22, Xingsha Road, Changsha, 410010 Hunan Province People’s Republic of China

## Abstract

**Background:**

Sepsis may be accompanied by acute respiratory distress syndrome (ARDS) in patients admitted to intensive care units (ICUs). It is essential to identify prognostic biomarkers in patients with sepsis and ARDS.

**Objective:**

Determine whether changes in the level of serum fibroblast growth factor 21 (FGF21) can predict the 28-day mortality of ICU patients with sepsis and ARDS.

**Methods:**

Consecutive sepsis patients were divided into two groups (Sepsis + ARDS and Sepsis-only), and the Sepsis + ARDS group was further classified as survivors or non-survivors. Demographic data and comorbidities were recorded. The Sequential Organ Failure Assessment (SOFA) score and serum levels of cytokines and other biomarkers were recorded 3 times after admission. Multiple Cox proportional hazards regression was used to identify risk factors associated with 28-day mortality in the Sepsis + ARDS group. Multivariate receiver operating characteristic curve analysis was used to assess the different predictive value of FGF21 and SOFA.

**Results:**

The Sepsis + ARDS group had a greater baseline SOFA score and serum levels of cytokines and other biomarkers than the Sepsis-only group; the serum level of FGF21 was almost twofold greater in the Sepsis + ARDS group (P < 0.05). Non-survivors in the Sepsis + ARDS group had an almost fourfold greater level of FGF21 than survivors in this group (P < 0.05). The serum level of FGF21 persistently increased from the baseline to the peak of shock and death in the non-survivors, but persistently decreased in survivors (P < 0.05). Changes in the serum FGF21 level between different time points were independent risk factors for mortality. No statistical difference was observed between the AUC of FGF21 and SOFA at baseline.

**Conclusion:**

A large increase of serum FGF21 level from baseline is associated with 28-day mortality in ICU patients with sepsis and ARDS.

**Supplementary Information:**

The online version contains supplementary material available at 10.1186/s12931-021-01778-w.

## Background

Sepsis is defined as a life-threatening organ dysfunction caused by the host’s deregulated response to an infection [1]. Sepsis may lead to acute respiratory distress syndrome (ARDS), a condition characterized by acute, diffuse, and inflammatory lung injury that leads to increased non-hydrostatic extravascular lung water, reduced lung compliance, and severe hypoxemia [2].

Despite therapeutic innovations, the mortality rate of patients with sepsis is as high as 25–30% [3] and the mortality rate of patients with ARDS can reach 35–45% [4]. Sepsis and ARDS are closely related, and most of these patients receive care in intensive care units (ICUs). Up to 50% of sepsis patients admitted to ICUs develop ARDS [5], and the mortality rate of patients with sepsis and ARDS is greater than that of patients with sepsis alone or ARDS alone [6, 7]. Thus, it is essential to identify the characteristics of sepsis patients who develop ARDS after ICU admission.

Sepsis and ARDS are heterogeneous inflammatory syndromes, and certain phenotypes might be associated with clinical outcomes. Several studies reported that sepsis patients with hyper-inflammation had higher mortality rates [6, 7]. Similarly, some studies showed that patients with ARDS and hypo-inflammation might have better clinical outcomes than those with ARDS and hyper-inflammation [8, 9]. There is also evidence that some pro- and anti-inflammatory biomarkers have prognostic value in patients with sepsis and ARDS [10, 11]. Previous studies suggested that biomarkers for ARDS can be classified as epithelial markers (surfactant proteins [SPs], Krebs von den Lungen-6 [KL-6] protein, vascular endothelial growth factor [VEGF], and soluble receptor for advanced glycation end-products [sRAGE]); endothelial markers (angiopoietin-2 [Ang-2]); and inflammatory cytokines (IL-1β and TNF-α) [12]. However, few studies directly examined the pro- and anti-inflammatory markers of patients with sepsis and ARDS. It is also uncertain whether the levels of pro- and anti-inflammatory cytokines are associated with the development of ARDS in patients with sepsis. Thus, it is necessary to identify novel serum biomarkers that can be used to classify disease severity in patients with sepsis and ARDS.

Fibroblast growth factor 21 (FGF21) is a peptide hormone that regulates energy homeostasis and glucose-lipid metabolism that is synthesized by the liver, adipocytes, pancreas, and brain [13]. The plasma level of FGF21 is greater in patients with metabolic disorders, such as type 2 diabetes, obesity, and mitochondrial diseases, and the level also increases during aging [14, 15]. Patients with inflammatory reactions, such as sepsis, pancreatitis, and systemic inflammatory response syndrome (SIRS), have increased serum levels of FGF21 [16, 17]. FGF21 apparently reduces oxidative stress by decreasing the level of reactive oxygen species (ROS), repressing nuclear factor kappa B (a pro-inflammatory factor), and reducing apoptosis [18–21]. Siahanidou et al. and our previous study demonstrated that FGF21 has value as a prognostic indicator in patients with sepsis [22, 23].

However, the relationship of FGF21 with prognosis in patients with sepsis and ARDS is uncertain. In this prospective cohort study, we aimed to evaluate whether changes in the serum level of FGF21 predict 28-day mortality of ICU patients who have sepsis and ARDS.

## Patients and methods

### Patients

This prospective cohort study included 231 consecutive patients with sepsis who were admitted to the Department of Critical Care Medicine of Changsha of Traditional Chinese Medicine Hospital (Hunan, China) between January 2019 and December 2020. Sepsis was diagnosed according to the Sepsis-3 criteria [1] and ARDS was diagnosed according to the Berlin definition [2]. All included patients were older than 18 years. The exclusion criteria were cancer, hospitalization or receipt of antibiotics during the preceding 2 weeks, and re-admission to the ICU.

All eligible patients were divided into a Sepsis-only group (patients who met the Sepsis-3 criteria and had no evidence of ARDS) and a Sepsis + ARDS group. The Sepsis + ARDS group was further divided into a survival group (patients who survived 28 days after initial diagnosis) and a non-survival group (patients who died within 28 days after diagnosis).

Demographic data (including age and gender) and medical history (including cardiovascular diseases, diabetes mellitus, chronic renal failure, and chronic obstructive pulmonary disease) were recorded. Laboratory values, hemodynamic parameters, ventilator settings, and medications were recorded at disease inception, during the worst period of disease, and during the recovery period. Sequential Organ Failure Assessment (SOFA) scores were calculated. The main clinical outcome measure was 28-day mortality.

Each patient received standard treatment during the ICU stay. This study was approved by the Ethics Committee of Changsha of Traditional Chinese Medicine Hospital and informed consent was obtained from all patients or their guardians.

### Cytokine measurements

Blood samples were collected within 24 h after diagnosis, during the worst period of disease, and 2–3 days prior to death or discharge (blood samples were collected from the patients every two to three days to monitor for all biochemical markers in our center). The serum levels of FGF21, interleukin 6 (IL-6), IL-10, tumor necrosis factor α (TNFα), procalcitonin (PCT), and C reactive protein (CRP) were determined using enzyme-linked immunosorbent assays (ELISAs) from Abcam (USA) according to the manufacturer’s instructions. Routine blood tests and blood gas analyses were performed in the hospital’s central laboratory.

### Statistical analysis

The normality of the distribution of a quantitative variable was tested using the Kolmogorov–Smirnov test (P > 0.10). Normally distributed data are expressed as means and standard deviations (SDs), and non-normally distributed data as medians and interquartile ranges (IQRs). Qualitative data are presented as number (%). For continuous variables, two-group comparisons were performed using Student’s *t*-test or the Mann–Whitney U test, depending on the normality of the distribution. A two tailed P value less than 0.05 was considered statistically significant. For categorical variables, the Chi-square test, Fisher’s exact test, or McNemar’s test was used as appropriate. The Hotelling T^2^ test was used to identify the significance of changes in the level of serum FGF21 and other markers.

To identify the risk factors for 28-day mortality of patients in the Sepsis + ARDS group, multiple Cox proportional hazards regression analyses were performed to measure the effects of different variables at three key times: (1) ICU admission; (2) peak of shock; and (3) before death or ICU discharge. The value of each measured parameter (P) is expressed as “P_t_”, where ‘t’ is the measurement time (1, 2, or 3); the change in a parameter between two times is expressed as “ΔP_t1–t2_”, where ‘t1’and ‘t2’ are the measurement times; and the percentage change in a parameter between two times is expressed as “∆P%_t1–t2_”_._

Multivariate receiver operating characteristic curve analysis was performed to compare the different value of FGF21 and SOFA detected at admission as a predictor for 28-day survival. All statistical analyses were performed using SPSS version 24.0 software (IBM SPSS, USA). A two tailed P value below 0.05 was considered significant.

## Results

### Comparison of the Sepsis and Sepsis + ARDS groups

We initially compared the clinical characteristics of patients in the Sepsis-only and the Sepsis + ARDS groups (Additional file 1: Table S1). The Sepsis + ARDS group had a greater respiratory rate, a lower PaO_2_/FiO_2_, a greater SOFA score, and higher levels of lactate (LAC), procalcitonin (PCT), C-reactive protein (CRP), interleukin 6 (IL-6), tumor necrosis factor α (TNFα), IL-10, and FGF21 (all P < 0.05). The serum level of FGF21 was almost two-times greater in the Sepsis + ARDS group.

### Comparison of survivors and non-survivors in the Sepsis + ARDS group

The survivors and non-survivors in the Sepsis + ARDS group had similar age, gender, and underlying diseases (Table 1). At baseline, the non-survivors had a significantly lower mean arterial pressure, hematocrit, and Glasgow Coma Scale (GCS) score; greater heart rate, body temperature, and SOFA score; and greater serum levels of creatinine, LAC, PCT, CRP, IL-6, TNFα, IL-10 (Table 1; all P < 0.05). The serum level of FGF21 was almost four-times greater in the non-survivors (Table 1; all P < 0.05).

**Table 1:** Baseline clinical characteristics of patients in the Sepsis + ARDS group who were survivors and non-survivors

Parameter	Non-survivors (n = 42)	Survivors (n = 119)	P value
Age (years)	68 ± 13	65 ± 16	**0.137**
Gender (male/female)	23/19	84/35	**0.062**
Coronary heart disease	13 (31.1%)	38 (31.9%)	**0.907**
Hypertension	12 (28.6%)	36 (30.2%)	**0.838**
Type 2 diabetes	5 (11.9%)	13 (10.9%)	**0.862**
Chronic renal failure	3 (7.1%)	8 (6.7%)	**0.926**
COPD	12 (28.6%)	39 (32.8%)	**0.615**
Length of stay	10 (2–22)	9 (3–21)	**0.578**
Mean arterial pressure (mmHg)	68 (35–116)	87 (48–120)	** < 0.001**
Respiratory rate (per min)	24 (12–40)	22 (13–38)	0.125
Heart rate (bpm)	110 ± 33	98 ± 20	**0.022**
Temperature (°C)	38.1 ± 1.4	37.2 ± 0.8	**0.019**
White blood count (10^9^/L)	15.0 ± 6.6	14.3 ± 7.2	0.682
Platelet count (10^9^/L)	131 (17–364)	178 (35–473)	0.055
Hematocrit (%)	31.3 ± 7.0	35.4 ± 5.7	0.050
PaO_2_/FiO_2_ (mmHg)	152.1 ± 55.7	164.6 ± 54.5	**0.021**
Total bilirubin (μmmol/L)	19.5 (4.6–124.7)	19.0 (4.8–98.5)	0.183
Creatinine (μmmol/L)	144.0 (44.9–1477.9)	96.4 (33.7–507.6)	**0.009**
Lactate (mmol/L)	3.7 (0.9–18.0)	2.7 (1.2–5.6)	** < 0.0001**
GCS score	9 (3–15)	11 (3–15)	** < 0.0001**
SOFA score	11 (3–21)	6 (2–18)	** < 0.0001**
CRP (mg/L)	134.9 (18.6–468.0)	100.7 (9.8–200.0)	** < 0.0001**
PCT (ng/mL)	16.96 (1.70–200.00)	3.07 (0.15–200.0)	** < 0.0001**
IL-6 (pg/mL)	367.7 (37.3–498.2)	89.6 (2.9–462.6)	** < 0.0001**
TNF-α (pg/mL)	63.9 (11.1–215.4)	12.3 (5.8–102.1)	** < 0.0001**
IL-10 (pg/mL)	597.6 (68.0–798.7)	108.6 (10.7–657.9)	** < 0.0001**
FGF21 (pg/mL)	3574.7 (327.3–6978.5)	986.6 (32.3–6121.3)	** < 0.0001**

*COPD* chronic obstructive pulmonary disease

### Dynamics of FGF21 in the Sepsis + ARDS group

**Fig. 1:**
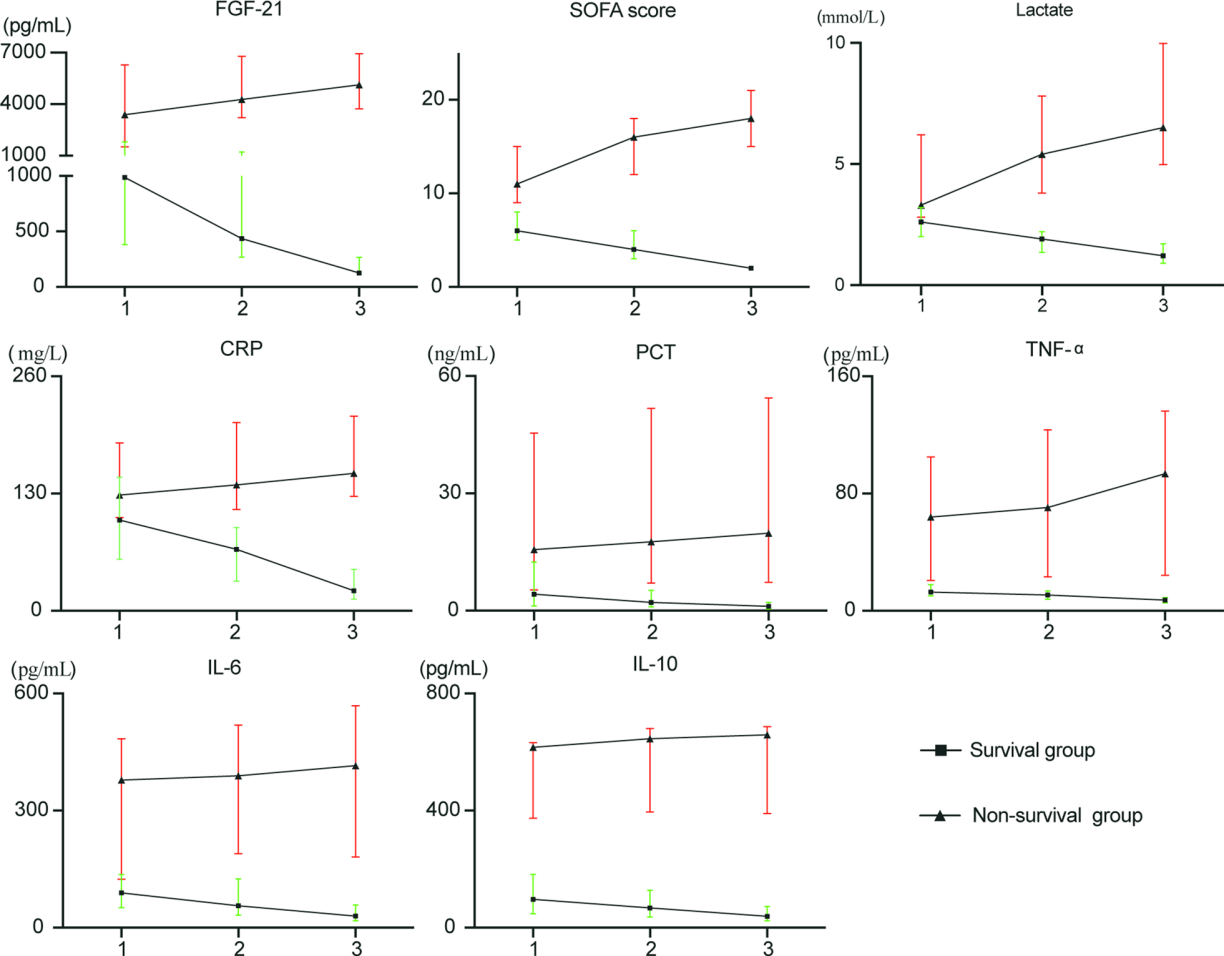
Levels of different laboratory and clinical parameters in survivors and non-survivors of Sepsis + ARDS at ICU admission (time-1), peak of shock (time-2), and before death or ICU discharge (time-3)

We recorded the changes of cytokines and other biomarkers while patients in the Sepsis + ARDS group were in the ICU (Fig. 1). The serum level of FGF21 persistently increased from baseline in the non-survivors, but gradually decreased in the survivors (both P < 0.05). In addition, pro-inflammatory biomarkers (CRP, PCT, TNF-α, IL-6,) and SOFA score also increased in the non-survivors, and decreased in the survivors (all P < 0.05).

### Risk factors for 28-day mortality in Sepsis + ARDS patients based on measurements at 3 crucial time points

We initially analyzed variables associated with 28-day mortality based on measurements at 24–48 h after ICU admission (Table 2). Before analysis, main parameters were changed into categorical variables according to their quartile values at baseline (Additional file 2: Table 2). The results indicated that serum FGF21 level, SOFA score, and serum level of CRP were independent risk factors for 28-day mortality (all P < 0.05).

**Table 2:** Cox proportional hazard analysis of baseline factors associated with mortality

Variable	Univariate Cox model		Multivariate Cox model	
	HR (95%CI)	P value	HR (95%CI)	P value
**SOFA**	**2.973 (2.043–4.327)**	** < 0.0001**	**3.507 (1.770–6.948)**	** < 0.0001**
**FGF21**	**3.388 (2.266–5.066)**	** < 0.0001**	**11.224 (3.237–38.917)**	** < 0.0001**
IL-6	2.437 (1.769–3.358)	< 0.0001	NA	0.639
TNF-a	3.071 (1.926–4.898)	< 0.0001	NA	0.051
IL-10	3.220 (1.977–5.792)	< 0.0001	NA	0.991
PCT	1.787 (1.339–2.385)	< 0.0001	NA	0.602
**CRP**	**1.011 (1.008–1.015)**	** < 0.0001**	**2.029 (1.305–3.155)**	**0.002**
LAC	1.944 (1.462–2.584)	< 0.0001	NA	0.062

Here and below: *NA* not applicable

We then analyzed variables associated with 28-day mortality based on initial measurements and measurements at the peak of shock (Table 3). The results indicated that the ΔSOFA_2–1_, FGF21_2_, and ΔFGF21%_2–1_ were independent risk factors for 28-day mortality (all P < 0.05).

**Table 3:** Cox proportional hazard analysis of factors at baseline (1) and the peak of shock (2) that were associated with mortality in the Sepsis + ARDS group

Variable	Multiple Cox model	
	HR (95% CI)	P value
**SOFA**_**1**_	**1.247 (1.096–1.418)**	**0.001**
SOFA2	NA	0.956
∆SOFA_2–1_	NA	0.692
∆SOFA%_2–1_	NA	0.617
FGF21_1_	NA	0.053
**FGF21**_**2**_	**1.001 (1.000–1.001)**	** < 0.0001**
**∆FGF21%**_**2–1**_	**15.269 (1.622–143.712)**	**0.017**
LAC_1_	NA	0.730
LAC_2_	NA	0.652
∆LAC%_2–1_	NA	0.812

Here and below: the difference in the value of a parameter (P) at different times is expressed as “ΔP_t1–t2_”, where ‘t1’ and ‘t2’ are the measurement times; and the percentage change in a parameter at different times is expressed as ∆P%_t1–t2_

We also analyzed variables associated with 28-day mortality based on measurements performed at 2–3 days before death or ICU discharge, measurements at the peak of shock, and initial measurements (Table 4). The results indicated that ΔSOFA_3–1_, ΔFGF21%_3–1_, ∆ LAC%_3–1_, and baseline CRP_1_ were independent risk factors for 28-day mortality (all P < 0.05).

**Table 4:** Cox proportional hazard analysis of factors at baseline (1), peak of shock (2), and at death or ICU discharge that were associated with mortality in the Sepsis + ARDS group

Variable	Multiple Cox model	
	HR (95% CI)	P value
SOFA_1_	NA	0.664
SOFA_2_	NA	0.735
**SOFA**_**3**_	**1.148 (1.023–1.289)**	**0.019**
∆SOFA%_2–1_	NA	0.399
∆SOFA%_3–2_	NA	0.955
∆SOFA%_3–1_	NA	0.625
FGF21_1_	NA	0.294
FGF21_2_	NA	0.197
FGF21_3_	NA	0.384
∆FGF21%_2–1_	NA	0.272
∆FGF21%_3–2_	NA	0.413
**∆FGF21%**_**3–1**_	**25.760 (1.482–447.610)**	**0.026**
LAC_1_	NA	0.838
LAC_2_	NA	0.834
LAC_3_	NA	0.830
∆LAC%_2–1_	NA	0.435
∆LAC%_3–2_	0.693	0.693
**∆LAC%**_**3–1**_	**1.512 (1.041–2.196)**	**0.030**
**CRP**_**1**_	**1.008 (1.003–1.014)**	**0.003**

### The predictive value of FGF21 for differentiating non-survivors and survivors in patients with Sepsis + ARDS

**Fig. 2:**
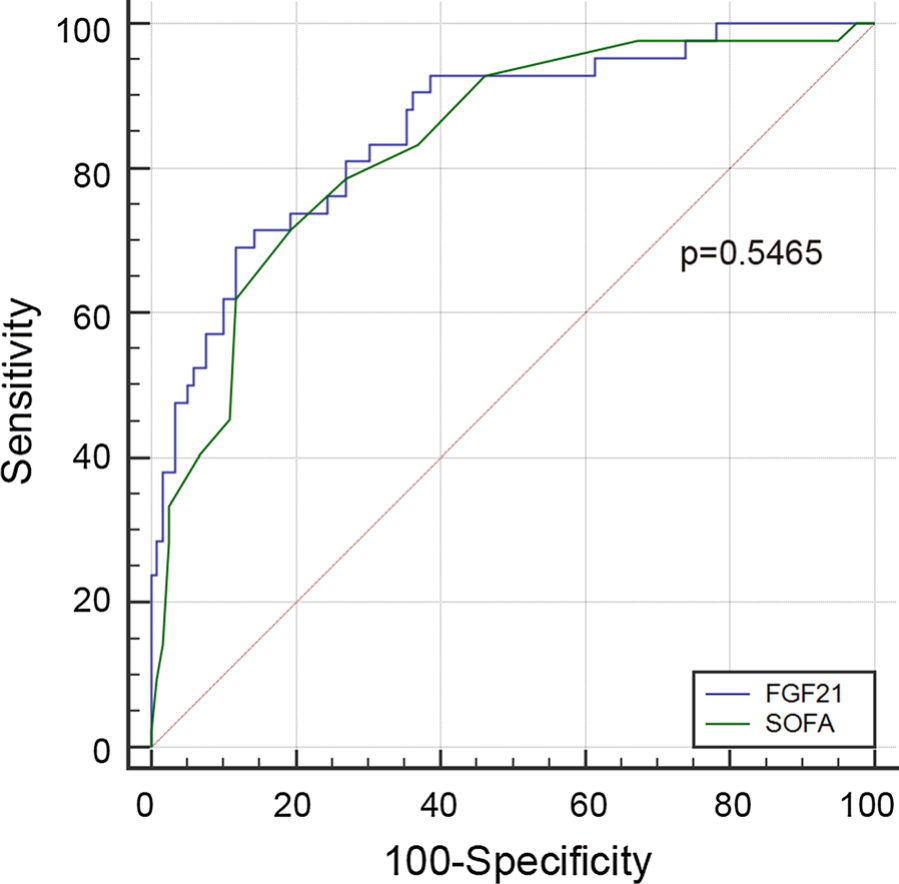
Receiver operating characteristic curve (ROC) of FGF21 vs. SOFA in predicting 28-day mortality of critical patients with sepsis + ARDS

We examined the predictive values of FGF21 and SOFA at baseline for differentiating non-survivor and survivor patients with Sepsis + ARDS by use of receiver operating characteristic (ROC) curve analysis (Table 5 and Fig. 2). The results showed that the AUC of FGF21 was 0.856, better than that of SOFA (0.832), but this difference was not statistically significant. Besides, the optimal cutoff was 2542 pg/mL for FGF21 level to better discriminate survivors from non-survivors, with a sensitivity of 69.1% and specificity of 88.2%. Thus, FGF21 is a promising biomarker for differentiating non-survivors and survivors among patients with Sepsis + ARDS.

**Table 5:** Accuracy of FGF21 and SOFA in differentiating non-survivors and survivors among patients with Sepsis + ARDS

Parameter	Areas under curve (AUC)	95%CI	Sensitivity (%)	Specificity (%)	Positive predictive value (%)	Negative predictive value (%)
FGF21	0.856^※^	0.792–0.906	69.1	88.2	68.2	87.5
SOFA	0.832	0.766–0.887	71.43	80.7	61.3	86.8

^※^Comparison of AUC between FGF21 and SOFA, P value = 0.5465

## Discussion

In this study, we found that the serum FGF21 level at baseline was greater in ICU patients who had sepsis and ARDS than in those who had sepsis alone. Furthermore, non-survivors in the Sepsis + ARDS group had persistently increasing levels of FGF21 during their ICU stays, but survivors in the Sepsis + ARDS group had persistently declining levels. Notably, our Cox proportional hazards model indicated that an increase in the serum level of FGF21 from the baseline was a significant risk factor for 28-day mortality in the Sepsis + ARDS group. To our best knowledge, this is the first study to identify the prognostic value of dynamic changes in the serum level of FGF21 in patients with sepsis and ARDS.

Previous studies demonstrated that the serum level of FGF21 was greater in adults and newborns who had sepsis [22, 23]. Our previous study demonstrated that the serum level of FGF21 was positively associated with serum inflammation biomarkers, including PCT, CRP, IL-6, and TNFα [23]. Patients with sepsis and ARDS have a greatly increased inflammatory state. Thus, it is not surprising that the serum level of FGF21 was greater in patients with sepsis and ARDS.

Elevation of inflammatory and anti-inflammatory biomarkers can occur concurrently during the early phase of sepsis [24]. Previous studies reported that FGF21 had anti-inflammatory effects during sepsis [25]. The present study, which compared patients with sepsis alone *vs.* sepsis and ARDS, identified much higher levels of serum FGF21 and pro-inflammatory factors (PCT, CRP, IL-6, and TNFα) in the Sepsis + ARDS group. This suggests a more intense interaction between pro-inflammatory and anti-inflammatory factors in patients who have sepsis and ARDS.

Immune cells function in the early stage of ARDS, so the focused biomarkers for ARDS in this stage are inflammatory cytokines, such as IL-1β and TNF-α which are mainly related to exudation and migration of inflammatory. However, the anti-inflammatory cytokines also play a critical role in this stage and should not be ignored. Besides it is also critical to identify novel biomarkers for late stage of ARDS which is not well investigated currently. FGF21 has anti-inflammatory and anti-oxidative stress effects [18–21]. This motivated our interest in the relationship between the dynamics changes of FGF21 and the prognosis of ARDS during sepsis in the present research.

In this study, we found that FGF21 is a promising prognostic biomarker for differentiating non-survivors and survivors among patients with Sepsis + ARDS (Table 1). Our analysis of disease progression in patients with sepsis and ARDS indicated the non-survivors had continuously increased serum levels of FGF21, and this was accompanied by persistent increases in the levels of multiple pro-inflammatory cytokines. In contrast, the survivors had gradually decreased levels of FGF21 and of pro-inflammatory cytokines. The presence of higher levels of pro- and anti-inflammatory biomarkers in the non-survivors is consistent with the presence of increased physiological stress.

Because the dynamic changes of pro- and anti-inflammatory markers appeared to be of vital importance in the progression of patients with sepsis and ARDS, we were interested in the following question: Which parameters can be used to predict the mortality of patients with sepsis and ARDS? Thus, we used Cox regression analysis to assess the prognostic value of biomarkers that were measured at admission to the ICU, at the peak of shock, and before death (non-survivors) or before ICU discharge (survivors). Our measurements at admission indicated that increased FGF21, LAC, and SOFA score were associated with poor prognosis in patients with sepsis and ARDS. This result is consistent with results from our previous study [23].

Our measurements at the peak time of shock (typically 1 week after ICU admission) indicated that the level of FGF21 at the peak did not show significant difference, but the percentage increase in the FGF21 from baseline (ΔFGF21%_2–1_) value had a much greater HR (15.269 [95% CI 1.622–143.712] vs. 1.247 [95% CI 1.096–1.418]). In other words, based on measurements at baseline and the peak of shock, we identified the percentage increase of FGF21 as the best predictor of poor prognosis in patients with sepsis and ARDS. The FGF21 at the peak may serve as a good predictor with enlarged sample size. The large changes in the serum level of FGF21 from admission to the peak of shock reflect the dynamics of pro- and anti-inflammatory factors in patients with sepsis and ARDS.

We also analyzed the relationship of the final clinical measurements (before death or ICU discharge) with patient prognosis. We found that the ΔFGF21%_3–1_, ΔLAC%_3–1_, and ΔSOFA_3–1_, and CRP_1_ were significant prognostic factors. Notably, ΔFGF21%_3–1_ was best predictor of poor prognosis (HR = 25.760 [95% CI 1.482–447.610]). This confirms that a large increase in the serum level of FGF21, either between the first and second measurements or between the first and third measurements, was strongly associated with 28-day mortality in patients with sepsis and ARDS. At the physiological level, this may be attributed to the anti-inflammation, anti-apoptotic, and anti-oxidative effect of FGF21 during sepsis.

## Limitations

First, this was a single-center study, and the total number of patients with sepsis and ARDS was relatively small. However, we have been pursuing this topic and collecting the records of sepsis patients for several years. A large and multi-center study should be performed to verify our observations. Second, because pro-inflammatory and anti-inflammatory factors both increased during sepsis, it is possible that other unmeasured anti-inflammatory cytokines might have also played a crucial role. We are currently analyzing the prognostic value of other anti-inflammatory biomarkers in sepsis patients, and plan further comparisons of their functions in patients with sepsis and ARDS. Third, some sepsis patients experience a hypo-inflammation phenotype. Most of our patients experienced hyper-inflammation, and this may be attributable to our exclusion criteria, such as exclusion of patients with cancer. The effect of changes in the serum level of FGF21 on the prognosis of patients with sepsis and ARDS who have hypo-inflammation is a topic that needs further study.

## Conclusion

An increase in the serum level of FGF21 after ICU admission of patients who have sepsis and ARDS is associated with a significantly increased risk of 28-day mortality.

## Supplementary Information

**Additional file1: Table S1.** Baseline clinical characteristics of patients in the Sepsis + ARDS group and the Sepsis-only group.

**Additional file2: Table S2.** Quartiles of △SOFA, Lac, FGF21, PCT, TNF-α, IL-6, IL-10, and CRP used in the Cox regression analysis.

## Data Availability

Data will not be shared with a reason.

## Abbreviations

ARDS: Acute respiratory distress syndrome
ICUs: Intensive care units
FGF21: Fibroblast growth factor 21
SOFA: Sequential Organ Failure Assessment
IL-6: Interleukin 6
IL-10: Interleukin 10
TNFα: Tumor necrosis factor α
PCT: Procalcitonin
CRP: C reactive protein
LAC: Lactate
GCS: Glasgow Coma Scale
ROC: Receiver operating characteristic curve
