# Supplementary material for: Does an increase in serum FGF21 level predict 28-day mortality of critical patients with sepsis and ARDS?
Source: Respir Res. 2021 Jun 22;22:182. doi: 10.1186/s12931-021-01778-w (PMC8216835; doi:10.1186/s12931-021-01778-w)
Supplement: Supplementary file 1 — Additional file1: Table S1. Baseline clinical characteristics of patients in the Sepsis + ARDS group and the Sepsis-only group. [file 12931_2021_1778_MOESM1_ESM.docx]

**Table S1. Baseline clinical characteristics of patients in the Sepsis+ARDS group and the Sepsis-only group.^*^**

| **Parameter** | **Sepsis+ARDS**  **(n=161)** | **Sepsis-only (n=69)** | **P value** |
| --- | --- | --- | --- |
| Mean arterial pressure (mmHg) | 80±19 | 89±15 | 0.106 |
| Respiratory rate (per minute) | 24(12~48) | 20(12~30) | **0.006** |
| Heart rate (bpm) | 106±25 | 97±12 | 0.068 |
| Temperature (°C) | 37.5±1.3 | 37.4±0.4 | 0.791 |
| White blood count (10^9^/L) | 14.4(0.26~54.24) | 13.37(3.29~42.35) | 0.690 |
| Platelet count (10^9^/L) | 168(17~527) | 135(55~420) | 0.367 |
| Hematocrit (%) | 32.0±6.8 | 32.0±5.7 | 0.432 |
| PaO_2_/FiO_2_ (mmHg) | 172.3±60.8 | 348.8±135.4 | **<0.001** |
| Total bilirubin (μmmol/L) | 18.4(4.0~124.7) | 17.6(7.3~83.35) | 0.151 |
| Creatinine (μmmol/L) | 99.9(33.7~1477.9) | 76.2(32.9~684.6) | 0.079 |
| LAC (mmol/L) | 2.7(0.5~18.0) | 2.1(0.7~5.6) | **<0.001** |
| GCS score | 11(3~15) | 11(3~15) | 1.000 |
| SOFA score | 7(2~21) | 4(2~13) | **<0.001** |
| CRP (mg/L) | 113.1(9.77~468.0) | 71.4(6.8~498.0) | **<0.001** |
| PCT (ng/mL) | 6.57(0.15~200.0) | 1.89(0.05~21.0) | **0.002** |
| IL-6 (pg/mL) | 113.4(2.93~498.1) | 56.7(4.0~345.6) | **0.001** |
| TNF-α (pg/mL) | 14.9(5.8~215.4) | 11.9(5.9~46.3) | **<0.001** |
| IL-10 (pg/mL) | 154.6(10.7~798.7) | 76.9(7.1~496.8) | **0.009** |
| FGF21 (pg/mL) | 1108.3  (32.3~6978.5) | 533.0  (41.1~3652.5) | **<0.001** |

*Here and below: Values are expressed as mean ± SD or median (IQR); ARDS, acute respiratory distress syndrome; bpm, beats per minute; GCS, Glasgow coma scale; SOFA, sequential organ failure assessment; PCT, procalcitonin; CRP, C-reactive protein; IL, interleukin; LAC, lactate; TNF, tumor necrosis factor; FGF, fibroblast growth factor.
