# Supplementary material for: Does an increase in serum FGF21 level predict 28-day mortality of critical patients with sepsis and ARDS?
Source: Respir Res. 2021 Jun 22;22:182. doi: 10.1186/s12931-021-01778-w (PMC8216835; doi:10.1186/s12931-021-01778-w)
Supplement: Supplementary file 2 — Additional file2: Table S2. Quartiles of △SOFA, Lac, FGF21, PCT, TNF-α, IL-6, IL-10, and CRP used in the Cox regression analysis. [file 12931_2021_1778_MOESM2_ESM.docx]

**Table S2. Quartiles of △SOFA, Lac, FGF21, PCT, TNF-α, IL-6, IL-10, and CRP used in the Cox regression analysis.**

| Variable | Quartile | Range |
| --- | --- | --- |
| △SOFA | 1 | 2–5 |
|  | 2 | 6–7 |
|  | 3 | 8–10 |
|  | 4 | 11–21 |
| Lac (mmol/L) | 1 | 0.7–2.1 |
|  | 2 | 2.2–2.8 |
|  | 3 | 2.9–4.3 |
|  | 4 | 4.4–18 |
| FGF21 (pg/ml) | 1 | 32.2–667.6 |
|  | 2 | 667.7–1453.6 |
|  | 3 | 1453.7–5653.1 |
|  | 4 | 5653.2–6978.5 |
| PCT (ng/ml) | 1 | 0.2–1.9 |
|  | 2 | 2.0–6.6 |
|  | 3 | 6.7to 17.1 |
|  | 4 | 17.2–200 |
| TNF-α (pg/ml) | 1 | 5.8–11.3 |
|  | 2 | 11.4–15.0 |
|  | 3 | 15.1–28.1 |
|  | 4 | 28.2–215.4 |
| IL-6 (pg/ml) | 1 | 2.9–56.2 |
|  | 2 | 56.3–113.44 |
|  | 3 | 113.5–331.4 |
|  | 4 | 331.5–498.2 |
| IL-10 (pg/ml) | 1 | 10.7–59.3 |
|  | 2 | 59.4–154.6 |
|  | 3 | 154.7–478.8 |
|  | 4 | 478.9–798.7 |
| CRP (mg/L) | 1 | 9.8–83.2 |
|  | 2 | 83.3–113.0 |
|  | 3 | 113,1–163.1 |
|  | 4 | 163.1–468.0 |
